# Single chamber permanent epicardial pacing for children with congenital heart disease after surgical repair

**DOI:** 10.1186/s13019-016-0439-6

**Published:** 2016-04-12

**Authors:** Tao Zhang, Yiwei Liu, Chengwei Zou, Hao Zhang

**Affiliations:** Department of Cardiac Surgery, Provincial Hospital Affiliated to Shandong University, Jinan, China; Center for Pediatric Cardiac Surgery, National Center for Cardiovascular Diseases and Fuwai Hospital, Beijing, China; Department of Cardio-Thoracic Surgery, Shouguang People’s Hospital, Shouguang, China; Peking Union Medical College and Chinese Academy of Medical Sciences, 167 Beilishi Road, Beijing, 100037 P.R. China

**Keywords:** Congenital heart disease, Permanent epicardial pacemaker, Iatrogenic atrioventricular block

## Abstract

**Background:**

To analyze the 10-year experience of single chamber permanent epicardial pacemaker placement for children with congenital heart diseases (CHD) after surgical repair.

**Methods:**

Between 2002 and 2014, a total of 35 patients with CHD (age: 26.9 ± 23.2 months, weight: 9.7 ± 5.6 kg) received permanent epicardial pacemaker placement following corrective surgery. Echocardiography and programming information of the pacemaker, as well as major adverse cardiac events (MACE) as heart failure or sudden death, were recorded during follow-up (46.8 ± 33.8 months).

**Results:**

Acute ventricular stimulation threshold was 1.34 ± 0.72 V and no significant increase was observed at the last follow-up as 1.37 ± 0.81 V (*p* = 0.93). Compared with initial pacemaker implantation, the last follow-up didn’t show significant increases in impedance (*p* = 0.327) or R wave (*p* = 0.635). Four patients received pacemaker replacement because of battery depletion. 7/35 (20 %) of patients experienced MACE. Although the age and body weight were similar between patients with and without MACE, the patients with MACE were with complex CHD (100 % vs.55.6 %, *p* = 0.04).

**Conclusion:**

High-degree iatrogenic atrioventricular block was the primary reason for placement of epicardial pacemaker for patients with CHD after surgical repair. Pacemaker placement with the steroid-eluting leads results in acceptable outcomes, however, the pacemaker type should be optimized for the children with complex CHD.

## Background

Permanent epicardial pacemaker have been utilized in clinical practice for several decades and the numbers of patients who require them are increasing. Pediatric pacemaker implants comprise < 1 % of all pacemaker implantations [1]. The main indication for permanent pacemaker implantation in children is high-degree congenital atrioventricular block (AVB) with a frequency of about 1/2000 [2, 3]. Another important indication is high-degree AVB following open-heart surgery, occurring with an incidence of 1 %. It is the main reason for placement of permanent epicardial pacemaker after congenital heart surgery [4]. Although endocardial pacemaker have several advantages, such as longer battery life, better sensing and pacing thresholds, epicardial pacemakers are more suitable for infants and younger children who are still under somatic growth, especially for patients with complicated cardiac deformities [5, 6]. However, epicardial pacemakers are less durable and have a higher incidence of complications as compared to endocardial pacemakers [7]. With the development of steroid-eluting leads appears to offer a better alternative to epicardial pacemaker. Its performance and longevity have been demonstrated to be comparable with the conventional endocardial leads [8].

Because of low economic statuses and undeveloped insurance systems, the single chamber pacemakers are utilized in many heart center for sick children in China. The current study aimed to review and analyze our 10-year experience in placement of single chamber permanent epicardial pacemaker and its mid-term outcomes.

## Methods

### Patient characteristics

A total of 44 CHD patients aged < 8 years received placement of epicardial pacemakers between 2002 and 2014 in our hospital. Nine children were excluded because they were associated with congenital AVB and pacemaker was implanted during the surgical repair. Thirty-five patients received permanent pacemaker implantation for the iatrogenic AVB (age: 26.9 ± 23.2 months, weight: 9.7 ± 5.6 kg). The study was approved by the ethics committee of Fuwai Hospital. Patient data were reviewed retrospectively. The diagnoses of CHD were summarized in Table 1 and all the patients received the primary corrective operation.

**Table 1:** Patient diagnoses

	ccTGA	DORV	TECD	TOF	VSD	ASD	Total
Iatrogenic AVB	8	6	4	4	11	2	35

*ccTGA* corrected transposition of great arteries, *DORV* double outlet right ventricle, *TECD* complete endocardial cushion defect, *TOF* tetralogy of Fallot, *VSD* ventricular septal defect, *ASD* atrial septal defect

### Implantation procedure

All procedures were performed under general anaesthesia. Steroid-eluting bipolar epicardial pacing leads were inserted into the diaphragmatic surface of the right ventricular free wall through the previous median sternotomy, and then connected to the plus generator within the subrectus pocket. The patients received pacemaker placement at 26 ± 3.1 days post-operation.

### Pacemaker parameters and pacing mode

Acute ventricular stimulation threshold, impedance, R wave, and pacing mode were recorded at implantation and at the last follow-up.

### Echocardiographic assessment

Left ventricular ejection fraction (LVEF) and left ventricular end diastolic diameter (LVEDD) were recorded at pre-implantation (1 week before pacemaker implantation), post-implantation (1 week after pacemaker implantation), and the last follow-up.

### Follow-up

The duration of follow-up was 46.8 ± 33.8 months. Pacemaker device-related complications were collected, including lead failure, elevated pacing threshold, poor sensing, and pocket infection. Age, body weight and type of cardiac deformities were compared between patients with and without major adverse cardiac events (MACE), which included heart failure or sudden death.

### Statistical analysis

Statistical calculations were carried out using SPSS version 19.0. Continuous data were presented as mean ± standard deviation or median and range. Categorical data were expressed as frequency and percentage. The independent sample t-test and one way analysis of variance were performed to compare the normally distributed variables when variances were homogeneous. If variances are not homogeneous, Dunnett’s T3 test would be performed. For categorical data, a comparison was performed using the Chi-square test and Fisher’s exact test. The Kaplan-Meier method was used to study the battery longevity and patients survival curve. A *P*-value lower than 0.05 was considered statistically significant.

## Results

### Early postoperative results

The operation time for pacemaker implantation (skin to skin time) was 119.1 ± 33.5 min. After surgery, 11 patients were extubated in the operation room and the rest were extubated within 4.8 h (range, 1.2–66 h). No blood transfusions occurred in the perioperative period. Except for a patient that suffered early postoperative death, who died from the low cardiac output syndrome after surgical repair for complex congenital heart disease, all patients were discharged from the hospital.

### Device characteristics

All the patients received a single chamber pacemaker with VVI pacing mode. The mode wasn’t changed during follow-up. Table 2 reported the data for ventricular stimulation threshold, impedance, and R wave.

**Table 2:** Analysis of the data for ventricular stimulation threshold, impedance, and R wave

	Intraoperation	Last follow-up	*P* value
Ventricular stimulation threshold(V)	1.34 ± 0.72	1.37 ± 0.81	*p* = 0.933
Impedance (Ω)	366.7 ± 88.0	331.9 ± 95.9	*p* = 0.327
R wave(mV)	12.3 ± 3.5	11.4 ± 4.9	*p* = 0.635

### Echocardiographic findings

**Fig. 1:**
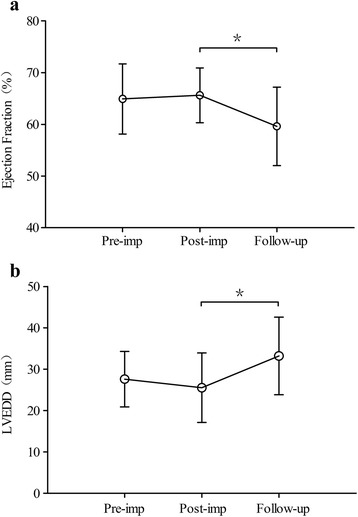
The tendency of LVEF and LVEDD at pre-implantation, post-implantation and the last follow-up: **a** *Left* ventricular ejection fraction:^*^ *p* = 0.03. **b** *Left* ventricular end diastolic diameter:^*^ *p* = 0.005

Compared with the post-operation, LVEF was significantly decreased in the last follow-up (65.6 ± 5.3 % vs. 59.6 ± 7.6 %, *p* = 0.03), while a significant increase was observed in LVEDD (25.5 ± 8.4 mm vs. 33.3 ± 9.6 mm, *p* = 0.005). Figure 1 depicts the tendency of LVEF and LVEDD at pre-operation, post-operation, and the last follow-up.

### Follow-up

Four patients underwent pacemaker replacement at 50.8 months (range, 30–80 months) post-surgery due to battery depletion. No pocket infection or lead fracture occurred. Freedom from generator replacement was 94.1, 86.9, 77.2 and 61.8 % at 30, 36, 48, 57 months, respectively.

**Fig. 2:**
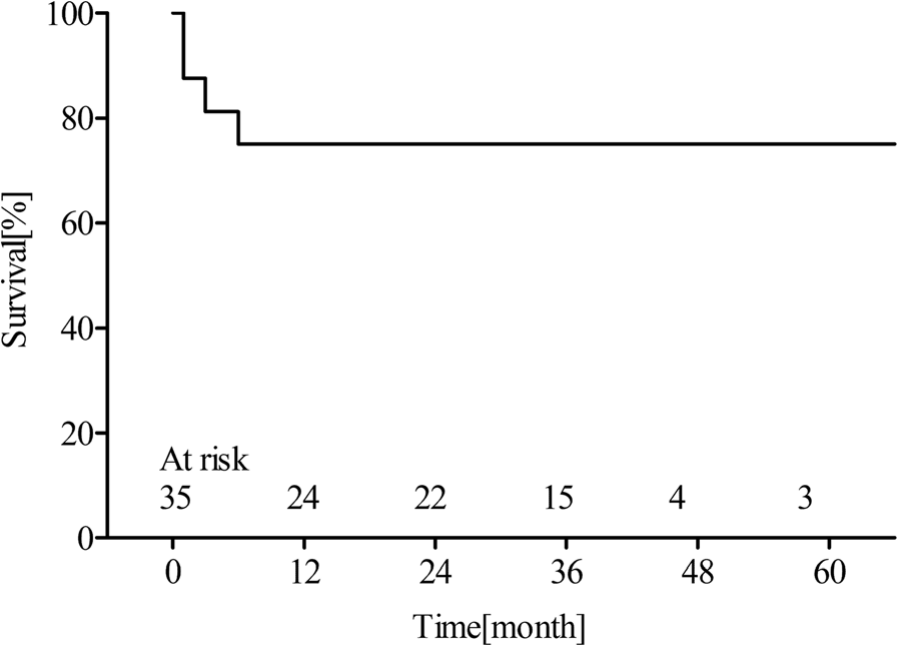
Estimated Kaplan-Meyer survival curve, which included the early mortality after pacemaker implantation

7/35 (20 %) of patients experienced MACE as heart failure or sudden death. Among them, four patients suffered from heart failure, with cardiac deformities of double outlet right ventricle (*n* = 2) and complete endocardial cushion defect (*n* = 2). Their conditions were improved after standard drug treatment. The other three patients suffered from sudden death at 6, 8, and 9 months post-operation, with cardiac deformities of complete transposition of great arteries (*n* = 1), double outlet right ventricle (*n* = 1), and tetralogy of Fallot (*n* = 1). The average age and weight of patients with MACE were 21.6 ± 30.66 months and 9.5 ± 6.4 kg, respectively. For patients without MACE, average age and weight were 20.4 ± 22 months and 9.8 ± 5.3 kg. There was no significant differences in age and body weight between patients with and without MACE (*p* > 0.05). But the type of cardiac deformities in patients with MACE were complex CHD (100 % vs.55.6 %, *p* = 0.046). Survival analysis of the patients is presented in Fig. 2.

## Discussion

CHD has become the leading type of birth defect in China. However, potential iatrogenic AVB is inevitable in a surgical operation and may result in postoperative pacemaker implantation. In our center, the primary reason for permanent pacemaker placement for children with CHD was iatrogenic AVB after congenital heart surgery. The most common type of cardiac deformities in patients with iatrogenic AVB is ventricle septal defect, followed by corrected transposition of great arteries (ccTGA), which was consistent with other developing counties such as Brazil and Egypt [9]. But in western countries, the most common type was complete transposition of the great arteries with ventricular septal defect, followed by complete endocardial cushion defect [10]. It is possibly because the surgeons in these countries prefer to perform corrective surgery on younger children with these two cardiac deformities, which could lead to a higher incidence of iatrogenic AVB.

For postoperative iatrogenic AVB, it is generally acknowledged that the proper duration of observation before permanent pacemaker placement is 7–14 days [11, 12]. However, in China, it is difficult for the patient’s family to accept postoperative pacemaker implantation, therefore, the observation time was extended, usually up to 3 weeks. Surgical placement of epicardial pacemaker is commonly known as the first choice for children with iatrogenic AVB who are younger than 4 years old, especially for the patient with complicated cardiac deformities, such as single ventricle and ccTGA.

All of our patients were implanted with a steroid-eluting lead. Compared with the traditional non-steroid-eluting lead, it could significantly reduce inflammation, with a low sensing threshold and is more durable [13, 14]. During follow-up, we didn’t observe a significant increase in the ventricular stimulation threshold, impedance, or R wave.

Generally speaking, children have a higher heart rate than adults, so their pulse generator is frequently exhausted earlier because of high pacing rates. In the patient less than 19 years old who had received permanent pacemaker implantation, it was reported that the mean longevity of the pacemaker generator was 5.5 years, and the lead was 10.8 years [6]. In the present study, no lead fracture was recorded, but four patients underwent reoperation for generator change because of battery depletion.

Although all patients’ cardiac functions were within the normal range (LVEF > 55 % is considered as normal [15]), the last follow-up examination showed significant decreases in LVEF compared with the post-operation examination. The major reason could be the pacing mode. Studies have shown that epicardial right ventricular free wall pacing could result in pathologic left ventricular dilatation and dysfunction, which could do harm to the patient’s cardiac functions [16, 17]. The significant risk factor for the development of LV dilatation and dysfunction was the presence of epicardial RV free wall pacing. One cross-sectional multicenter study showed that LV pacing especially apical and lateral wall pacing were associated with the best preservation of LV function, which appears to be related to preserved mechanical synchrony and contraction efficiency [15]. Surgical access to the LV is possible through the left lateral thoracotomy [18], therefore, the pacing site could be optimized in the future study.

Twenty percent of patients in our study had MACE as heart failure or sudden death. All these patients were iatrogenic AVB following complex congenital heart surgery. These patients received a single chamber pacemaker. Currently, dual chamber pacing systems are the first choice for pediatric patients in western countries, but single chamber pacing systems are still widely used in China. It is no doubt that dual chamber pacing is better than single chamber pacing, because it more closely resembles cardiac physiology [19]. Therefore, with improvements in the public health insurance system, for the complex CHD patient who suffered from iatrogenic AVB after surgical repair, dual chamber pacing may reduce morbidity from heart failure and sudden death.

## Conclusion

In summary, iatrogenic AVB following congenital heart surgery is the primary reason for the placement of permanent pacemaker in CHD patients. The epicardial approach for permanent pacemaker implantation provided acceptable outcomes for iatrogenic AVB. However, for the complex CHD patient with iatrogenic AVB, the pacemaker type should be optimized.

## Abbreviations

ASD: atrial septal defect
AVB: atrioventricular block
ccTGA: corrected transposition of great arteries
CHD: congenital heart diseases
DORV: double outlet right ventricle
LVEDD: left ventricular end diastolic diameter
LVEF: left ventricular ejection fraction
MACE: major adverse cardiac events
TECD: complete endocardial cushion defect
TOF: tetralogy of Fallot
VSD: ventricular septal defect
